# Parents’ views on care of their very premature babies in neonatal intensive care units: a qualitative study

**DOI:** 10.1186/1471-2431-14-230

**Published:** 2014-09-13

**Authors:** Gillian Russell, Alexandra Sawyer, Heike Rabe, Jane Abbott, Gillian Gyte, Lelia Duley, Susan Ayers, on behalf of the “Very Preterm Birth Qualitative Collaborative Group”

**Affiliations:** School of Psychology, University of Sussex, Brighton, BN1 9QH UK; Centre for Maternal and Child Health Research, School of Health Sciences, City University London, London, EC1R 1UW UK; Academic Department of Paediatrics, Brighton and Sussex University Hospitals Trust, Royal Alexandra Children’s Hospital, Eastern Road, Brighton, BN2 5BE UK; Bliss (The Special Care Baby Charity), 9 Holyrood Street, London Bridge, London, SE1 2EL UK; National Childbirth Trust, Alexandra House, Oldham Terrace, Acton, London, W3 6NH UK; Nottingham Clinical Trials Unit, University of Nottingham, Nottingham, NG7 2UH UK

**Keywords:** Preterm birth, Neonatal intensive care unit, Qualitative research

## Abstract

**Background:**

The admission of a very premature infant to the neonatal intensive care unit (NICU) is often a difficult time for parents. This paper explores parents’ views and experiences of the care for their very premature baby on NICU.

**Methods:**

Parents were eligible if they had a baby born before 32 weeks gestation and cared for in a NICU, and spoke English well. 32 mothers and 7 fathers were interviewed to explore their experiences of preterm birth. Although parents’ evaluation of care in the NICU was not the aim of these interviews, all parents spoke spontaneously and at length on this topic. Results were analysed using thematic analysis.

**Results:**

Overall, parents were satisfied with the care on the neonatal unit. Three major themes determining satisfaction with neonatal care emerged: 1) parents’ involvement; including looking after their own baby, the challenges of expressing breast milk, and easy access to their baby; 2) staff competence and efficiency; including communication, experience and confidence, information and explanation; and 3) interpersonal relationships with staff; including sensitive and emotional support, reassurance and encouragement, feeling like an individual.

**Conclusions:**

Determinants of positive experiences of care were generally consistent with previous research. Specifically, provision of information, support for parents and increasing their involvement in the care of their baby were highlighted by parents as important in their experience of care.

## Background

Preterm birth is the single most important determinant of adverse outcomes in terms of survival, quality of life, psychosocial and emotional impact on the family. Very preterm birth accounts for 1.4% of live births in the UK but 51% of infant deaths [1], and of those babies that do survive 5-10% develop cerebral palsy. Those without disability have a two-fold or greater increased risk for developmental, cognitive and behavioural difficulties [2, 3].

Preterm birth and subsequent hospitalisation of the baby are associated with psychological strain and emotional stress for the parents [4, 5]. The admission at birth of a very premature infant to the neonatal intensive care unit can be highly traumatic and distressing for parents [6]. Growing awareness of how difficult this experience can be for parents has contributed to significant changes to enhance family-centred care within Neonatal Intensive Care Units (NICU). Family centred care involves healthcare professionals actively considering parents’ experience of having a premature baby, and working within a policy framework to improve families’ experiences of care by changing the NICU environment to be more personalised and supportive of their needs [7–10].

Understanding of peoples’ experiences of healthcare services has improved considerably over recent decades [11, 12]. An increasing number of studies have used qualitative methods to explore what staff can do to help parents during their baby’s stay on the neonatal unit, and to highlight areas of care particularly important to parents [13]. For example, as part of The Parents of Premature Babies Project: Your Needs (POPPY Project), 55 interviews were conducted with parents of preterm babies about their experiences of care on the neonatal unit. Parents most valued consistent, clear information, receiving emotional support, and practical guidance and encouragement in caring for their baby [7]. Another study highlighted mothers’ anxiety regarding lack of knowledge of the neonatal equipment, wires and monitors [14]. Encouraging parents to spend more time with their infant and to actively participate in their care has also been shown to increase parental satisfaction [15]. One study reported that the baby’s medical condition was not related to parents’ assessment of care in the NICU; rather, the emphasis was on the personal and communication aspects of care [16]. Improving our understanding of what is important to parents of premature babies about their experiences of NICU may assist healthcare services in better supporting and caring for parents, and consequently their preterm infants. Few studies have explored fathers’ perceptions of care on NICU, which is essential in facilitating family centred care.

We conducted a qualitative study exploring parents’ experiences and satisfaction with care during and immediately after the birth of their very premature baby [17]. Studies have shown that interviews with patients provide a richer and often more realistic picture of the care they received in comparison to surveys [18]. Although parents’ evaluation of care in the NICU was not the aim of these interviews, all parents spoke spontaneously and at length on this topic. We therefore analysed parents’ experiences of NICU separately, and the results are reported here.

## Methods

A qualitative study of preterm birth using purposive sampling where all eligible parents whose baby was born at one of three hospitals in the previous six months were invited to take part. 32 mothers and 7 fathers agreed (26%) and were interviewed to explore their experiences of preterm birth. Recruitment took place between June 2011 and November 2011 at three tertiary care centres in South East England.

### Participants

Parents were eligible to take part if they had a baby born before 32 weeks gestation and spoke English well. They were eligible if at least one member of the couple wanted to participate or if they were single. Parents of babies who died were also included so that the sample also represented the experience of this group of parents of preterm babies.

### Design and procedure

Parents were recruited from three tertiary care centres in South East England by posters in the neonatal units or by letter of invitation. Letters were either posted or given to parents if they had been on the neonatal unit for longer than two weeks and met the eligibility criteria. Parents returned a card indicating their willingness to participate, and the study researcher then contacted them to discuss the study and arrange the interview. Before the start of the interview parents were given the opportunity to ask questions and informed that participation was entirely voluntary. Parents were also informed that any information provided would be confidential and anonymised. All parents gave written informed consent. All interviews were conducted by one of the authors (AS), who is a psychologist with experience of interviewing women in the perinatal period. Interviews lasted for approximately 45 minutes, and were recorded and then transcribed with all identifying information removed. Parents were interviewed separately in the cases where both the mother and father were participating, with the exception of two couples who specifically requested to be interviewed together. Interviews were concluded when no new information emerged and data saturation had been achieved.

### Materials

An interview schedule was used consisting of 12 open-ended questions. Questions related to parents’ experiences during the birth (e.g. can you describe what happened during the birth of your baby?) and their satisfaction with care (e.g. was there anything particular about the care you received, at birth and immediately after your baby was born, that you were particularly happy/unhappy with?) and covered topics such as who was involved at the birth and which aspects of care had a particular impact on parents. These results have been reported elsewhere [17, 19]. Parents were not asked direct questions about their experiences of neonatal care but all chose to discuss the topic spontaneously and in depth during the interview. As the interviewer had the freedom to follow a line of enquiry introduced by the parent, parents were encouraged to elaborate on their responses. A questionnaire was also administered to obtain socio-demographic information. Basic obstetric and neonatal data were collected from medical records.

### Data analysis

Inductive thematic analysis was used to carry out qualitative analysis of the interview transcripts in order to identify, analyse, and report themes and patterns within the data. Thematic analysis offers an accessible and theoretically flexible, as well as systematic, approach to analysing qualitative data [20]. In line with the thematic analysis method, transcripts were firstly read and re-read so as to become familiarised with the data, and initial codes of interest were generated. Initial codes were then organised into potential themes and relevant codes were collated under these themes. Following this, themes were reviewed in relation to the generated codes and the entire data set. Finally, themes were named and defined. NVivo Version 10 qualitative analysis software was used to sort codes and themes. For this report, direct quotes are coded (number = participant number; mother/father; D/C = baby discharged from hospital, NICU = baby in hospital, Dec. = baby deceased) to ensure anonymity.

### Ethical approval

The study received approval from the National Research Ethics Committee South East Coast – Kent. Reference: 11/LO/0143. Date of approval: 13th May 2011.

## Results

Between January and June 2011, 123 couples or single parents were approached and 39 (26%) returned cards indicating their interest in participating in the research (32 mothers and 7 fathers). Interviews were conducted in a quiet room either at the parent’s home (*n* = 34) or in the hospital (*n* = 5). Demographic and obstetric characteristics are given in Table 1. Parents were between 25 and 44 years old, the majority were White European and either married or cohabiting. Babies were born between 24 and 32 weeks gestation. The sample included two mothers whose babies had died following birth. Overall 84% of women reported pregnancy complications and 94% of babies had neonatal complications.

**Table 1:** **Demographic and obstetric characteristics of sample**

Parent details	*N* = 39 (%)
**Ethnicity**	
White European	29 (74)
Indian	3 (8)
Pakistani	2 (5)
Filipino	2 (5)
Other	3 (8)
**Marital status**	
Married/living with partner	37 (94)
Partner	1 (3)
Separated	1 (3)
**Education**	
None	2 (5)
GCSEs/O levels	9 (23)
A-levels/Diploma/City & Guilds	12 (31)
Undergraduate	6 (15)
Postgraduate	2 (5)
Professional	8 (21)
**Employed**	33 (85)
**Income** (*n* = 37)	
< £10,000	3 (8)
£10,000 - £19,999	7 (19)
£20,000 - £29,999	15 (41)
£30,000 - £39,999	6 (16)
> £40,000	6 (16)
**BIRTH DETAILS**	*N* = 32 (%)
**Gestation at birth (weeks)**	
31-32	11 (35)
30-31	3 (9)
29-30	3 (9)
28-29	3 (9)
27-28	4 (13)
26-27	4 (13)
25-26	1 (3)
24-25	3 (9)
**Type of birth**	
Vaginal	13 (40)
Caesarean	19 (60)
**Multiple birth**	11 (34)
**Parity**	
1	24 (75)
2	6 (19)
3	2 (6)
**Mean days on neonatal unit (** ***SD*** **and range)**	49.6 (25.1; 25-115)
**Baby on neonatal unit at time of interview**	6 (19)
**Mean time since birth (** ***SD*** **and range)**	154 days (57; 44-344)

Three major themes were identified as contributing to parents’ satisfaction with neonatal care: 1) parents’ involvement; 2) staff competence and efficiency; and 3) interpersonal relationships with staff. Table 2 displays an overview of the themes with quotes which illustrate the themes and the number of interviews in which these themes were mentioned. These themes are described below.

**Table 2:** **Themes, quotes, and number of interviews themes were mentioned**

Theme	Quotes	Number of interviews theme was in (*N*=37)
**Parents’ involvement**		
Looking after their own baby	*“[The neonatal unit] are great about you being able to open the incubator and they get you very involved in looking after them, and changing nappies, and cleaning bottoms and so you’re doing as much of the care as you can”. (12, mother, D/C).*	22
	*“I had lovely [name of nurse], who’s a great nurse, and she showed me how to change the nappies straight away which was all a bit fumbly with at first, and you’re very nervous”. (20, mother, NICU)*	
	*“It was a long time before we was allowed to change nappies. But once we were, we had to always ask permission. So she didn’t feel like ours for a very, very long time. It felt like this baby that I’d had but almost given away, if that makes sense”. (19, mother, D/C)*	
	“*And I think as a mum you do feel quite helpless at that point, because you think…‘this is my contribution to her care at the moment, because everyone else is having to care for her because she’s so tiny.’ So that really was quite good for me”. (1, mother, D/C)*	
The challenges of expressing breast milk	*“I kept asking, when do I start expressing, I I’m rubbing the, trying to get it to come out but nothing’s working um… and it was about day 4, I think before they said to me, oh yea, here’s a kit, go and express”. (21, mother, D/C)*	10
	“*Normally they say don’t express or breast-feed for about 3 days if you’ve had an emergency C-section and things like this and get time for the milk. Whereas the baby unit was literally from straight away ‘oh we need some breast milk, we need breast milk, we need breast milk,’ which put [name of mother] under excessive pressure and stress, because she felt like she was letting [name of baby] down, even though she wasn’t really ready herself to actually start for it”. (4, father, D/C)*	
Easy access	*“Yeah, I really appreciated [the diary]. They sent it back with all his stuff. And I thought that was really important, cos it’s hard not being able to go and see him. But to be able to call in afterwards and read this little diary was really nice”. (2, mother, D/C)*	14
	“*[The neonatal staff] appreciate that there’s a bond between parents and baby that needs to be maintained. The biggest plus was… the (neonatal) unit got us a room over the road”. (4, father, D/C)*	
**Staff competence and efficiency**		
Communication	*“It was a busy environment, and so if communication had been bad I would have said, ‘I can appreciate why it was bad’, but it wasn’t, it was really good, so communication for me was number 1. Absolute number 1. And that really helped, that felt, made us really reassured. It gave us confidence throughout the whole experience. Really good”. (1, father, D/C).*	19
	*“At [Hospital A], I used to have to keep checking the board, and I’m like ‘It says up there he’s having a scan. What’s he having a scan for?’ and they’re like ‘We dunno.’ And I’m like ‘What do you mean you don’t know?” (2, mother, D/C)*	
	*“Because you come in one day, say the day before, especially there was a guy there that, he promoted to hold her, literally whenever we was in, either of us, he would say, ‘Hold her, it’s the best thing you could do’. And then you’d come in the next day thinking ‘oh yes, I get to hold her’. And you have a different nurse that says, ‘no, no you’ve held her this week, you don’t need to hold her for the rest of the week’… and then you’d almost feel devastated that you couldn’t do that.” (19, mother, D/C).*	
	*“the other doctors had decided, that it was too soon and they needed to wait a bit longer, but nobody had told us that, so we’re expecting results, and we’re not getting anything we haven’t even had the test”. (21, mother, D/C)*	
Experience and confidence	*“…if there was someone on I’d think, ‘Oh yeah, I really like them, they seem to really know, you know, be on the ball’ and I felt confident. And I did feel very sort of reassured, erm, that she [the nurse] was very, you know no nonsense kind of like… very… came across very confident”. (6, mother, dec).*	17
	*“It was reassuring as well, because it was almost one-on-one care. So it was like she was being monitored the whole time. If she needed anything, there was somebody there straight away. Erm, so you felt that you could leave her, and there was nothing we could do, it was just the medical staff and they needed to do what they needed to do”. (1, father, D/C)*.	
	“*All the doctors that were there, as far as I’m concerned, were the experts. The doctors had, you know, been in this industry for like 15, 20, 25 years. They knew, they knew their stuff inside out you know, the information was never flaky…” (16, mother, D/C).*	
	*“And both of them [the nurses] said ‘We wouldn’t continue support.’ and I think you appreciate that because you want to know. They come with many, many years of experience and they’ve seen…babies in this situation”. (32, mother, dec)*	
	“95% of the women at [Hospital B] are lovely, you can’t fault them but if you’d ring up at night, and you’d get a certain nurse you’d go ‘Oh s**t’, because they just, they never kind of, seemed to be on the ball… It’s just your heart would sink if you got a certain nurse you’d go, ‘Oh god’, whereas if I knew I had a good nurse, I felt very confident. If I knew I had a not so good nurse, I’d be agitated’. (23, mother, NICU)	
Information and explanation	*“And I think they were really, you know, explained everything. Every time we went to the incubator, whoever the nurse was on looking after her, you know, always explained how she’d been doing, how she’d been…they talked…it was really lovely”. (6, mother, dec)*.	30
	“*She explained every machine to me: ‘That’s to monitor her heart rate’, she goes ‘Your baby’s not on oxygen, she’s on air just to help her lungs, 'cos you do know baby’s really small. Baby’s not sick mummy.’ And she explained everything to me. The machines, how they incubate her.’ (30, mother, D/C).*	
	*“Sometimes, I don’t know, things need to be explained a bit more, or like when they do the doctors' rounds, they go through all the birth details, just as a recap for everything, and there’s some things in there that it would be nice for that to be explained…I guess they do explain it to you when you first come in but they don’t.. you can’t remember, you can’t take stuff in. I think that follow up explanation of everything… cos it took me ages to ask…” (20, mother, NICU)*.	
	*“While the doctors are really good if you’ve got any questions, I shouldn’t have had to get a book myself out of the cupboard…It would be good if it, you know, there’s a set structure that everybody has to go through, everybody’s given the same information, needs to be put on the baby’s file, gone through x y and z with parents”. (22, mother, NICU)*	
**Interpersonal relationships with staff**		
Sensitive and emotional support	*“It was almost overwhelming how lovely everyone was and just, before during and after, the whole process just really sympathetic and, and came across like they were hurting too…[The staff were] incredibly empathic and you know even, they were giving us the, the prognosis with Z, and they had tears in their eyes you know the way that they were saying it and you kind of, it makes it feel like you’re a person who is experiencing a terrible thing rather than just another number going through the process”. (32, mother, dec)*.	16
	*“[The nurse] was quite patronising and she said um, because I was adamant I just wanted them to have breast milk, and she said ‘oh a little bit of formula won’t do them any harm’… She even said, she even said to me, ‘why didn’t you have a C-section it would have been a lot easier?’ This is when I was looking over [name of baby] and I was upset, really upset, I didn’t know if she was going to make it or not’. (15, mother, D/C)*	
	*“part of you thinks that the nurse is judging you 'cos you’re not there all the time”* (*24, mother, D/C*)	
	*They’re constantly talking to them, when they’re changing them and so on – ‘hello love,’ they talk to it… It’s not the case of just cleaning and feeding them. You’re talking to my baby, you’re making my baby reassured”. (2, father, D/C)*	
	*“I felt like she genuinely cared, she wasn’t just doing her job she genuinely cared um, and um, yeah bless her she was just amazing with him, really really good with him”. (31, mother, D/C)*	
	*“they just said 'look, you know she’s in safe hands, you know we love her to bits you can leave her here for as long as possible, for as long as you like'”. (12, Mother, D/C)*	
Reassurance and encouragement	*“She kept willing us forward. ‘Why don’t you try this? Why don’t you do his nappy?’ I was like ‘Oh no’, very scared, but very encouraged to touch him, talk to him… Things around us were very positive’. (3, mother, D/C)*.	16
	*“They’re focusing on the bright side of having a baby. They talked to me about that ‘Oh she must be lovely. She’s a girl, she must be, sort of like, have dark hair’. And it feels great that”. (10, mother, D/C)*	
Feeling like an individual	*“Yeah, I just found our experience very good, it was very, I suppose, personal in a sense. I wasn’t, I didn’t feel like a piece of meat. I felt like a human that was passed around and people were caring…they were really willing me on and I felt like a person that was going through this and I had support around me rather than: you’re in a hospital, you’re passed from buck to buck”. (3, mother, D/C)*	18
	*“also [nurse] was special because she treated [name of baby] like a person not like a patient, so she listened to what he was trying to tell us”. (31, mother, D/C)*	
	*“The doctors, I think 'cos obviously they’re quite stern and they just kind of come in and do what they have to do and then kind of leave, which is a shame. They never really know your child inside out, they just read the sheet on top…I’ve had some doctors come in going, ‘Well who’s this then?’, and I think well you’ve seen her 3 or 4 times you should know her name by now, silly little things like that just, to add the personal touch”. (23, mother, NICU)*	

**NB** Although 39 parents took part, the total number of interviews was 37 because two couples asked to be interviewed together.

### Parents’ involvement: *“if there’s stuff you can do, that is nice for you both”*

This theme centred around parents being able to participate in the care of their baby on the neonatal unit, and included: looking after their own baby, the challenges of expressing breast milk, and easy access to their baby.

#### Looking after their own baby

Twenty-two parents talked about the importance of being allowed to help with looking after their own baby, for example washing, cleaning and nappy changing, as well as being able to touch and hold their baby (12, mother, D/C). Parents appreciated being shown how to do these things by neonatal staff, and being present when they were done (20, mother, NICU*)*. Two parents mentioned the negative feelings that emerged as a consequence of not being allowed to help with personal care for their baby and missing out on performing parental duties (19, mother, D/C). Parents described how helping with personal care took on greater significance due to their feelings of helplessness about what was happening to their baby (1, mother, D/C).

#### The challenges of expressing breast milk

Ten parents described the importance of expressing breast milk in the care of their baby. While certain mothers spoke positively of a breastfeeding specialist who came round to help them, others referred to a lack of assistance from staff with expressing, as well as a lack of information regarding available facilities (21, mother, D/C). Parents also described feeling under considerable pressure from neonatal staff to produce breast milk immediately after the birth and in sufficient quantities (4, father, D/C).

#### Easy access

Fourteen parents mentioned that they appreciated neonatal staff facilitating as much access to their baby as possible, and their efforts to help strengthen parent-infant bonds. Units allowed twenty four hour access, and there were positive comments from parents who reported feeling welcome to visit or phone the neonatal unit any time of day or night. Staff also helped parents to develop a close relationship with their baby by taking photos, making a diary to illustrate the baby’s progress and giving parents a keepsake bag (2, mother, D/C).

A further way in which staff facilitated the bond between parent and baby was by securing a place at a house run by a charity which provides free ‘home away from home’ accommodation for families near hospitals (a facility only available at one of the sites). This enabled parents to stay close to their baby and maintain a degree of normal family life, as well as facilitating the regular provision of breast milk. Parents described this facility as “invaluable” and “a lifesaver”, and felt extremely grateful to the neonatal staff as a result of their efforts (4, father, D/C).

### Staff competence and efficiency*: “they seem to really… be on the ball and I felt confident”*

Nearly all parents made reference to the importance of staff displaying competence and efficiency in their role as clinicians. This theme included the following subthemes: communication, experience and confidence, and information and explanation.

#### Communication

Nineteen parents cited communication, both between neonatal staff themselves and with parents, as a major factor in determining their experience of the neonatal unit. Feeling that channels of communication were always open, especially given the busy environment of the neonatal unit, helped parents feel reassured and confident (1, father, D/C). Conversely, failures in communication led to parents’ lacking confidence in staff. Parents appreciated this could be partly due to the constant change over between shifts and lack of continuity of staff. A number of parents described not being informed if their baby had been moved or had experienced a sudden change in health, while others mentioned procedures or transfers happening without any apparent communication between staff (2, mother, D/C).

Just under a quarter of parents said being given conflicting advice and information from staff was confusing and stressful. While they understood that staff may have differences in personal opinion, parents felt that these issues should be discussed beforehand so they were not given inconsistent advice. For example, some nurses promoted kangaroo care, and therefore encouraged parents to hold their baby as much as possible, while others on the same ward were opposed to it and parents felt reprimanded for wanting to do this. This left some parents bewildered and in fear of doing the wrong thing. One set of parents describe how it felt to get their hopes up only to have them dashed the next day when a different member of staff had a conflicting opinion (19, mother, D/C). Another mother described how she and her husband were told that their baby had gone for some tests, but later found out that the doctors had decided to postpone these tests (21, mother, D/C).

#### Experience and confidence

Just under half of parents interviewed describe the importance of feeling that neonatal staff were, as several mothers put it, ‘*on the ball’*. Staff behaving in a confident manner reassured parents that the situation was under control, and that their child was receiving the best possible clinical care (6, mother, dec). Staff being vigilant and attentive by making frequent checks, taking notes every few minutes, maintaining cleanliness and regularly monitoring their baby was extremely important to parents. Witnessing efficiency and professionalism made parents feel more comfortable with leaving their baby in hospital (1, father, D/C). Feeling that some of the neonatal doctors and nurses were very experienced, dedicated to their job and highly knowledgeable was valued by several parents, as it gave them further confidence that their child was in expert hands (16, mother, D/C). For one mother who had to make the difficult decision about whether to turn her daughter’s life support machine off, feeling the staff were experienced helped her make this difficult decision (32, mother, dec).

While the majority of parents were positive about the clinical competence and capabilities of most of the staff, a number of parents expressed worries over a small proportion of the neonatal nurses. The perception of lack of competence among this minority of staff led to stress and made parents feel like they were not doing enough for their baby (23, mother, NICU).

#### Information and explanation

Thirty parents mentioned that they valued staff providing information and explaining medical details. Staff volunteering explanations and being able to answer questions reassured parents. Most parents wanted frequent updates on their baby’s health, and they also appreciated information about their baby’s daily routine (6, mother, dec). The way in which staff explained complicated, and sometimes distressing, medical details about the health and progress of their baby was also important to parents. Taking time to explain and using accessible language was valued by many parents (30, mother, D/C). However, some parents mentioned finding it difficult and overwhelming to try to take in all the information being given and felt that staff did not appreciate the sheer volume of information they had to take in. Consequently parents described being scared of missing or forgetting important information. Some were intimidated by the whole experience and therefore hesitant to ask questions (20, mother, NICU). There was a general feeling among some parents that ‘if you don’t know, you can’t ask’, especially concerning the long-term care of their baby. Some felt the information was not tailored for the parents of premature babies and, given the complexity of the situation, expressed a desire for a specific premature baby advisor. A number of parents described having to find out general information for themselves, either from posters in the neonatal unit, other parents or from pamphlets they happened to pick up (22, mother, NICU).

### Interpersonal relationships with staff*:*“*They make you feel like you*’*re a person who is experiencing a terrible thing rather than just another number”*

The majority of parents cited staff empathy as an important factor in their experience of the neonatal unit. This theme refers to the interpersonal interactions with the staff, such as sensitive and emotional support, reassurance and encouragement, and being made to feel like an individual. Even if staff were professional and clearly skilled at their job, it was the kindness and compassion of certain staff that often stood out in the minds of parents.

#### Sensitive and emotional support

Sixteen parents cited the kind and caring nature of the staff, and the emotional support they provided as positive aspects of NICU care. Some parents described it as like being in a family with the staff, a sense of ‘we’re all in this together’ , which made it easier for them to cope. A substantial number of parents commended the nurses for treating their role on the neonatal team as more than just a job; they felt that neonatal nurses went out of their way to provide emotional support and beyond their functional duties to care for both the parents and baby. Small acts of kindness such as providing cups of tea, common courtesies, and being polite and friendly were also much appreciated by parents. Parents spoke about the neonatal staff’s sensitivity to parents. Some nurses showed personal concern for the welfare of the parents, and encouraged parents to look after themselves. One of the parents whose baby died particularly praised the empathy of staff (32, mother, dec). Conversely, when parents experienced a lack of sensitivity or empathy from neonatal staff they found this distressing (15, mother, D/C). One mother described feeling judged for not being there all the time (24, mother, D/C).

Parents also highlighted how staff showed similar support and sensitivity to their babies. For example one father valued the nurses taking time to talk to the baby and provide comfort (2, father, D/C). Two mothers also described nurses that were particularly caring and loving to the baby (31, mother, D/C and 12, mother, D/C). For one mother this helped her leave her baby in the NICU when she had to go home.

#### Reassurance and encouragement

Sixteen parents mentioned receiving reassurance, encouragement and praise from staff as a beneficial experience in the NICU (3, mother, D/C). Parents understood that staff have to be realistic about the baby’s prognosis, but they appreciated their efforts to find the positive in a situation and build parents’ confidence back up during low moments (10, mother, D/C).

#### Feeling like an individual

Just under half of parents discussed being made to feel like an individual by staff. Parents valued feeling that their baby was important and treated as an individual. Staff knowing parents’ names and remembering details from previous conversations made parents feel like they were high on a list of priorities and receiving personalised care (3, mother, D/C).

Parents also valued the staff making the baby feel like a person, not a patient (13, mother, D/C). In contrast, feeling anonymous contributed to a negative experience of care for some parents, who expressed feeling disappointed that staff did not know them or their babies (23, mother, NICU).

## Discussion

This study identified three domains that were important factors in determining parents’ experiences with NICUs following very preterm birth. These were parents’ involvement in looking after their premature baby, staff competence and efficiency, and interpersonal relationships with staff.

The themes reported in the current study confirm the findings of previous qualitative and quantitative research. For example, previous studies have consistently identified parents’ involvement in the care of their preterm baby as extremely important to them [14, 21, 22]. Studies show that parents with infants hospitalised in NICUs are most worried about the alteration in their parental role e.g. [23]. Performing parental duties, such as feeding, changing and holding their baby, has been recognised as a means of parents connecting with their baby and taking on the role and identity of parents [6, 24–26]. A previous interview study with mothers also found that when mothers are involved in their child’s care they report a feeling of *“participation”* and more positive well-being [22]. The process of attachment can be delayed or disrupted after having a preterm baby and supporting this bond is particularly important as it has long-term consequences for the parent-infant relationship and the baby’s development [10]. Our findings also highlight the importance of supporting mothers in expressing and breastfeeding, and indicate how difficult this can be for some women and their partners. A previous qualitative study with mothers indicated that mother’s felt their main purpose during their baby’s stay was to express milk and the authors suggest a number of recommendations such as staff providing encouragement and support to breastfeeding mothers, providing a private room for breastfeeding and expressing, and referral to a breastfeeding counsellor for support where necessary [27].

Our finding of the importance to parents of communication is consistent with previous studies which found parental satisfaction to be highly dependent on the amount and quality of communication between the care providers and the parents [28–32]. Receiving conflicting advice and mixed messages from staff can heighten the anxiety of parents. This was highlighted by the POPPY Steering Group [7] who noted that, at a time of vulnerability, it was ‘crushing’ to follow one person’s advice and then feel criticised for doing so by another staff member.

Similarly, the importance of staff competence and of parents feeling assured by the vigilance, experience and knowledge of staff is consistent with previous research [8, 28]. Indeed a large-scale study of neonatal care found the best aspect of care to be the skill and experience of nurses [33]. Whilst most parents wanted as much information as possible, some felt overwhelmed by the sheer volume of information provided and struggled to process it all. Thus, it is crucial for staff to reinforce and repeat information, and allow time for parents to ask questions to clarify their understanding [34]. Moreover, whenever possible, it would seem important for staff to individually assess parent preferences and tailor the information-giving accordingly [16].

The majority of parents cited interpersonal relationships with staff as one of the most important factors affecting their satisfaction with the neonatal intensive care unit. Again, this is similar to previous studies [7, 16]. For example, in interviews with mothers, neonatal nurses who ‘chatted’ were singled out as those who truly made a difference to parents’ neonatal experience [29]. Some researchers maintain that neonatal nursing is a three way interactional process between nurse, mother, and baby, and nursing both the mother and infant facilitates the connection between the mother and baby [29, 35, 36]. It is also thought that social interactions with staff on a more personal level is important because it makes it less intimidating to ask questions and easier to express concerns [37]. This in turn facilitates parents’ ability to actively participate and take up their role of primary caregiver [37].

### Strengths

This study provides an in-depth insight into the experiences and satisfaction with care of parents of very preterm babies in the NICU. The inclusion of fathers and bereaved parents also provides a valuable and unique perspective of their experiences of the NICU. Trustworthiness was enhanced by the use of a well-established and appropriate form of analysis, ensuring that participants were given adequate opportunity to refuse participation in the study, the encouragement of a rapport between interviewer and interviewee, frequent debriefing sessions between the team members, and a discussion of results with peers who were not part of the research team.

### Limitations

These results should be considered keeping in mind that parents’ evaluation of care in the NICU was not the aim of these interviews. Therefore parents’ views on this topic might not have been explored fully.

One quarter of parents accepted the invitation to be interviewed, which is a good response for this type of study. However, the experiences reported in this study may not be applicable to all parents who give birth to a very premature baby. For example, there is evidence to suggest that there is a higher incidence of very preterm birth in certain ethnic groups [38] and in women from very deprived areas [39]. The current sample comprised of largely white, educated and married parents which suggests a possible self-selection bias (as parents responded to a letter of invitation only it was not possible to collect information about parents who did not accept our invitation to take part). Therefore more research is needed to explore if these finding are applicable to parents from different backgrounds. Furthermore, parents were recruited from three tertiary care sites in South East England and it is possible that perceptions of care could vary across levels of units and different locations.

The interviews were conducted between one month and almost a year after the birth, and as such, the experience may not have been at the forefront of the parents’ memories. However, the infants were on the neonatal unit for many weeks and such a significant and emotional experience is often remembered well [40]. Studies also show that satisfaction assessed early on may be particularly influenced by expectations, and as many very preterm births are unexpected this could have a negative impact on satisfaction ratings. In comparison, parents may report higher satisfaction post-discharge because of their baby’s improved condition compared to when they were first on NICU.

One other possible limitation of this study, as noted in other studies of patient satisfaction, is that parents might be reluctant to express critical comments about the care, a so-called ‘gratitude bias’ towards the staff who cared for their preterm baby [41]. This should have been minimised by the fact that the researcher carrying out the interviews was not involved in clinical care. However, the involvement of clinicians in identifying potential participants (the letter of invitation was signed by the consultant neonatologist) may have influenced who responded to the invitation to participate and may have led to a socially desirable response [41, 18]. Future studies should consider methods to remove this potential bias, such as letters being sent from someone not involved in the mother's or baby's care.

### Implications

Although not designed to directly explore parents’ experiences of the NICU, the findings of this study and other studies in the field, have a number of implications for clinical practice. Firstly, although our sample was not selected to be representative, the findings suggest there is considerable scope to improve family centred care. All staff can contribute to family centred care, and it is important that training is provided so that family centred care can be implemented across units. It is also crucial that parents are given the opportunity to provide feedback about their experiences of the NICU [10]. Secondly, since involvement in the care of their baby is important to parents, staff in NICUs should facilitate parent participation as much as possible in looking after their own baby [6]. Some parents described a diary that staff kept about their baby’s care, which helped keep parents informed when they could not be there. Practices like this could be more widely adopted across NICUs. Thirdly, clear and effective strategies should be employed to aid communication between staff and parents. For example, some hospitals have integrated notes kept by the side of the baby’s cot so that information is accessible to parents in clear language [10]. Another innovative approach is using technology such as BabyLink, a software program that produces reports summarising the baby’s clinical condition accessible to parents [42]. Providing audiotapes of parent-doctor consultations could be another way of improving parents’ satisfaction with communication [43].

## Conclusions

In summary, the present research provides an insight into the factors considered most important in determining parents’ experiences and satisfaction with neonatal care. While parents were largely satisfied with care, most felt there were aspects that could be improved to alleviate or reduce distress and anxiety. This study suggests that the most important determinants of parents’ satisfaction with NICU care are being able to perform parental tasks, proficient communication, good information provision and sensitive and emotional support. Understanding the experiences of parents as they try to cope with their premature baby can inform the development of family-centred practice in neonatal intensive care units [29].

## Authors’ information

GR worked on this study as part of her MSc. in Experimental Psychology at the University of Sussex.

AS is a Research Fellow at the Centre for Maternal and Child Health Research at City University London.

HR is a Senior Clinical Lecturer and Consultant Neonatologist at Royal Sussex County Hospital, Brighton.

JA and GG are representatives from parents groups (Bliss and NCT, respectively).

LD is the Chief Investigator of the NIHR Preterm Birth Programme.

SA is a Professor of Maternal and Child Health Research at City University London.
